# Driving down malaria transmission with engineered gene drives

**DOI:** 10.3389/fgene.2022.891218

**Published:** 2022-10-19

**Authors:** William T. Garrood, Piotr Cuber, Katie Willis, Federica Bernardini, Nicole M. Page, Roya E. Haghighat-Khah

**Affiliations:** ^1^ Department of Life Sciences, Imperial College London, London, United Kingdom; ^2^ Department of Molecular Biology, Core Research Laboratories, Natural History Museum, London, United Kingdom

**Keywords:** gene drive, homing, malaria, disease control, CRiSPR/Cas, mosquitoes

## Abstract

The last century has witnessed the introduction, establishment and expansion of mosquito-borne diseases into diverse new geographic ranges. Malaria is transmitted by female *Anopheles* mosquitoes. Despite making great strides over the past few decades in reducing the burden of malaria, transmission is now on the rise again, in part owing to the emergence of mosquito resistance to insecticides, antimalarial drug resistance and, more recently, the challenges of the COVID-19 pandemic, which resulted in the reduced implementation efficiency of various control programs. The utility of genetically engineered gene drive mosquitoes as tools to decrease the burden of malaria by controlling the disease-transmitting mosquitoes is being evaluated. To date, there has been remarkable progress in the development of CRISPR/Cas9-based homing endonuclease designs in malaria mosquitoes due to successful proof-of-principle and multigenerational experiments. In this review, we examine the lessons learnt from the development of current CRISPR/Cas9-based homing endonuclease gene drives, providing a framework for the development of gene drive systems for the targeted control of wild malaria-transmitting mosquito populations that overcome challenges such as with evolving drive-resistance. We also discuss the additional substantial works required to progress the development of gene drive systems from scientific discovery to further study and subsequent field application in endemic settings.

## 1 Introduction

### 1.1 The current landscape of malaria control

Collectively, mosquitoes are responsible for more human morbidity and mortality as well as economic losses than any other animal (Winegard, 2019). Malaria is transmitted during a female mosquito’s bloodmeal, causing an estimated 241 million cases and 627,000 deaths globally in 2020, with 95% of cases and 96% of malaria deaths taking place in the African continent, and mostly (∼80%) in children under 5 years old (WHO, 2021a). The deadliest malaria parasite in humans is *Plasmodium falciparum* (Welch, 1897), which is the most prevalent in the African continent, representing a major public health threat in endemic areas.

The malaria parasite is transmitted by approximately 30–40 *Anopheles* mosquito species with wide geographic ranges that extend beyond malaria-endemic regions, posing a risk of re-introduction of disease in areas where malaria has been eliminated (CDC - Centers for Disease Control and Prevention, 2020). The *Anopheles gambiae sensu lato* species complex contains the most significant mosquito vectors in sub-Saharan Africa, consisting of at least nine morphologically indistinguishable sibling species, some of which are sympatric (*i.e*., occur within overlapping geographical areas) (Coluzzi et al., 1979; Favia et al., 1997; Hunt et al., 1998; Coetzee, 2004; O’Loughlin, 2020).

Following a period of tremendous progress in malaria elimination owing to mass insecticide use, bed nets and improvements in clinical management, the last century has witnessed the introduction, establishment and expansion of mosquito-borne diseases into diverse new geographic ranges (Randolph and Rogers, 2010). Socio-demographic changes (Doumbe-Belisse et al., 2021)—resulting in more densely populated urban areas, substandard housing and other anthropogenic modifications to the environment–as well as emerging parasite drug resistance, the vector’s insecticide resistance and rising global mean temperature, have all contributed to their dramatic resurgence (Hemingway et al., 2006). The predicted further expansion of malaria towards higher elevations and more temperate areas, particularly densely populated regions with no prior exposure to the pathogens, signifies outbreak risks where public health systems are unlikely to be prepared (Colón-González et al., 2021).

Disrupting the transmission chain of malaria requires the reduction of one or more of the closely interlinked factors: the pathogen, the host’s susceptibility, and the vector. Existing tools—*e.g*., long-lasting insecticide-impregnated mosquito bed nets, indoor residual spraying, artemisinin-based combination therapy and removal of larval habitats—have saved millions of lives (WHO, 2021a). Malaria infection numbers and mortality rates were declining steadily for a decade until a sobering report in 2017 revealed that this progress had stalled (WHO, 2017). This was in part owing to the emergence of mosquito insecticide and antimalarial drug resistance. In 2020 there was an estimated increase of 69,000 worldwide malaria deaths from the previous year, of which approximately two thirds of the additional deaths were associated with disruptions in the provision of malaria prevention, diagnosis and treatment during the COVID-19 pandemic (WHO, 2021a). The COVID-19 pandemic has highlighted worldwide health inequalities (Jensen et al., 2021) and strong evidence indicates that the uptake of malaria control measures is particularly limited amongst individuals with low socio-economic status, whilst malaria also inhibits economic growth, increasing the vulnerability of the poorest (Worrall et al., 2005). Meanwhile, ongoing conflict and instability in parts of malaria-endemic areas, such as Democratic Republic of the Congo, Nigeria (Ren, 2019), and more recently, Mali, is also thought to have contributed to disruptions in malaria control programmes.

To get back on track and accelerate progress against malaria, improved and more equitable access to malaria control is crucial. Innovation and new prevention tools are also key to fast-track progress. The World Health Organisation’s approval and recommendation of the first of its kind malaria vaccine, Mosquirix RTS,S in October 2021 for children most at risk of the deadliest malaria parasite, *P. falciparum,* represents an opportunity for high impact. After over 30 years in development, the vaccine holds great promise as a public health tool, with an expected overall reduction of ∼30% severe malaria cases and significant reductions in overall hospital admissions due to malaria in children (Laurens, 2020).

Another compelling breakthrough is the development of the novel genetic control strategies discussed below to complement existing Integrated Vector Management (IVM) activities based on ecological, economic, and social criteria tailored to the requirements of an endemic region. IVM has been an essential part of vector control activities, which incorporates surveillance, public relations, and education as well as the use of specific conventional methods such as insecticides and biological control to reduce the targeted mosquito species. The success of IVM based on these conventional control methods relies heavily on the ability of humans to seek out and access the insect vectors’ habitats. Some breeding sites are inaccessible, cover a wide area, and are subject to repeated flooding, and may act as a possible source of re-colonisation of local anopheline populations that helps drive the seasonality of malaria transmission (Clennon et al., 2010).

The mosquito’s naturally evolved mate seeking behaviour can be exploited to introduce novel genetic traits into a target population to reduce disease transmission. If the released mosquitoes carry a genetic trait that renders their progeny unable to transmit disease, then the wild mosquito population can be changed to be refractory to disease known as the population replacement (or population modification) strategy (Alphey et al., 2020; WHO, 2021b). Another example of the population replacement strategy is the reversal of insecticide resistance through the spread of insecticide-susceptibility effectors (Kaduskar et al., 2022). If the released mosquito instead passes on a gene that eliminates or reduces the disease-transmitting portion of their progeny (e.g., *via* a sterility or lethal gene targeting only female progeny), the population can be reduced over time, also breaking the cycle of disease, known as the population suppression strategy (Alphey et al., 2020; WHO, 2021b). These types of efforts are termed genetic control.

The ability to genetically modify (i.e., transform) insects is fundamental to a practical genetic control strategy. Once this was achieved the potential for controlling the transmission of mosquito-borne disease with genetically modified mosquitoes was openly discussed and a long-term plan was formulated (Meredith and James, 1990; WHO, 1991; James, 1992). Since then, mosquito transformation technologies have proved to be powerful tools for genetic analysis and manipulation and have led directly to improvements in both suppression and replacement genetic control strategies. For example, the RIDL strategy, release of insects carrying a dominant lethal (Thomas et al., 2000), has been genetically engineered in the Dengue/Zika mosquitoes, *Aedes aegypti* (Phuc et al., 2007) for the population suppression strategy and eliminates the need for irradiation used in the sterile insect technique (SIT), which aims to reduce the ability of a target species to produce viable offspring. RIDL strains carry a tetracycline-repressible lethal genetic system that can be reared on tetracycline in the laboratory; their offspring cannot survive to adulthood in its absence. The strategy was first implemented in the Cayman Islands, and showed a population reduction of approximately 80% (Alphey et al., 2010; Harris et al., 2011). The Mendelian inheritance patterns of RIDL strains means that repeated releases are required, even following suppression (though at a lower level), to counter resurgence of the mosquito population (Carvalho et al., 2015).

There is also usually a fitness cost associated with genetically modifying mosquitoes, and as a result there are selective pressures towards the loss of transgenes (Catteruccia et al., 2003; Irvin et al., 2004; Marrelli et al., 2006; Lambrechts et al., 2008; Oye et al., 2014). Therefore, a system is required to spread the desirable trait (effector) through the wild vector populations at a greater than Mendelian rate, even if they confer a selective disadvantage (such as impaired fertility or sterility), to such levels that pathogen transmission no longer occurs, thus breaking the cycle of disease and reducing its burden or eliminating it altogether. A “gene drive” is a mechanism designed to show non-Mendelian patterns of inheritance where more progeny inherits the selfish gene than Mendelian inheritance would predict. This enables the frequency of the desirable trait to increase in the population despite having an associated fitness cost (Burt, 2003).

Consequently, the success of a gene drive mechanism is pivotal to fix introduced effector genes (transgenes) into wild populations. The World Health Organization (WHO) released a guidance framework for testing genetically modified mosquitoes, which aims to ensure that modified mosquitoes are already evaluated during product development to ensure that they are effective, competitive and any “risks (taking) into account both the likelihood and the magnitude of harm that may occur from a specific action, particularly with regard to national protection goals” are identified and reduced to acceptable levels ahead of their release (WHO, 2021b).

Significant progress has been made in the lab development of a suite of engineered gene drive systems in insects (Raban et al., 2020; Wang et al., 2021), each with varying characteristics suited to diverse contexts (Section 1.2). Due to the breadth of genetic approaches each with diverse characteristics of persistence and spread (depending on the transgenic component and its behaviour), and the broad range of conditions under which they might be used, as well as the diverse nature of the receiving environment, it is not possible to provide a general set of characteristics that could be applied to all gene drive technologies. Instead, as with other tools, case-by-case testing will be required to understand the advantages and disadvantages of a particular gene drive approach, where the potential risks and benefits are taken into consideration. Frameworks for risk assessment and regulation have been developed at various levels (NASEM, 2016; James et al., 2018, 2020; Teem et al., 2019), and in 2021, the revised version of the Guidance framework for testing genetically modified mosquitoes was published by the World Health Organization, which takes into account recent technical progress made in this rapidly advancing field (WHO, 2021b).

To date, many gene drive systems have been developed exclusively in the model organism, *Drosophila*, except for CRISPR-based homing drives, which have also been tested in proof-of-principle and multigenerational experiments in malaria mosquitoes. Homing drives encode an endonuclease (such as CRISPR/Cas9) that recognises and cuts a target DNA sequence of ∼12–40bp in the host genome, which when repaired by homology-directed repair (HDR) using the drive allele as a template, will convert a hemizygous cell into a homozygous for the gene drive (Burt, 2003). Recent advances in the development of this gene drive system are in part due to the use of well-characterised meiotic promoters in *An. gambiae* to drive the expression of easily transferable CRISPR/Cas9 components.

In this review, we examine lessons learnt from the development of these CRISPR-based homing endonuclease gene drives that are essential for the development of effective gene drive systems that overcome challenges such as with evolving drive-resistance and make an impact in the wild malaria-transmitting mosquito populations. We also discuss the additional substantial work needed for the technology to progress from the scientific discovery labs to further preparatory study and to subsequent application of the technology in malaria-endemic settings.

### 1.2 Self-sustaining vs. self-limiting gene drives

Self-sustaining gene drives, such as those based on a homing mechanism (Burt, 2003), maintain themselves between generations by causing alleles to increase in frequency in the target population every generation, in some cases to fixation (Alphey et al., 2020). These are expected to spread very quickly where there are limited fitness costs associated with the gene drive element. A single release could theoretically result in the spread of the desired trait into all populations of the same species, which is beneficial to applications such as malaria prevention that seeks to affect all target wild-populations (Noble et al., 2018), particularly in areas that are difficult to access.

However, there is notable uncertainty around the extent to which entire population conversion with self-sustaining gene drives is actually possible due to natural genetic variation between populations and the possibility of the emergence of resistance (Hammond et al., 2017). Modelling of self-sustaining gene drives that aim to suppress the target population demonstrated a likely significant (e.g., 90%) reduction in the number of adult mosquito vectors in the rainy season, but did not always find total elimination (Eckhoff et al., 2017). This is due to seasonal effects on the wild vector populations or the insufficient efficacy of the desired trait (Eckhoff et al., 2017). In addition, lab-developed mosquito colonies are likely to have some fitness costs compared to wild vector populations despite introgression to a local genetic background, which may also limit their spread.

For some potential population replacement applications, such as where confinement to the target area is preferred, gene drives that are limited by space, “spatially restricted (or localised) drives”, such as engineered underdominance, are purposefully designed to have threshold-dependent invasion dynamics. These are expected to spread the desired trait only when the gene drive element is introduced above a particular frequency and would be spatially limiting if migration into geographically non-target populations occurs rarely and below the drive threshold (Davis et al., 2001; Altrock et al., 2010, 2011; Marshall and Hay, 2012). Threshold (or frequency)-dependent gene drives (such as underdominance, which is a high-threshold gene drive) might therefore be considered more controllable, though it should not be assumed that such drives would remain localised, and some level of preparedness [such as the realistic worst case scenario (RWCS) approach (Connolly et al., 2022)] and monitoring for possible gene drive spread into populations of non-target organisms should be considered (Backus and Delborne, 2019). Population replacement could also be limited by time, “temporally limited”, such as in the daisy-chain system (Noble et al., 2019) or the killer-rescue system (Gould et al., 2008; Edgington and Alphey, 2018), which eventually declines and is eliminated from the population due to fitness effects. These aspects may be useful for understanding the spread dynamics of a gene drive system during the early phases of testing because these systems are theoretically easily reversible, such as through the release of wild type mosquitoes for the reversal of threshold-dependent gene drive systems. In malaria mosquitoes, self-eliminating gene drive components (autonomously driving Cas9) have recently been tested that could present an alternative approach to selectively removing components that are no longer needed from target populations (Ellis et al., 2022). However, threshold-dependent gene drives have not yet been developed in malaria mosquitos, and if they were, they would require longer-term repeated releases when below the drive threshold, resulting in increased costs associated with their mass production and release.

### 1.3 Homing-based gene drives

Homing endonucleases are a class of “selfish” genetic elements. They induce double strand breaks in DNA at specific recognition sites. This activates the cell’s recombination repair mechanism using the homologous chromosome carrying the homing endonuclease gene (HEG) as a template (*i.e*., homology directed repair: HDR), thereby spreading the inheritable HEG throughout the population (Goddard et al., 2001). Windbichler et al. (2011) showed the successful use of this system a decade ago where a synthetic homing endonuclease gene (I-SceI) under the control of germline regulatory regions significantly increased its transmission to the progeny in caged transgenic population of the human malaria mosquito *An. gambiae*.

The use of HEGs in natural mosquito populations depends on the ability to re-engineer their specificity towards native mosquito sequences to disrupt genes essential for its vectorial capacity or viability, or to introduce anti-pathogenic genes at selected loci. The re-engineering of natural HEGs to target endogenous genes in the mosquito genome has proven challenging, with only a few notable examples (Chan et al., 2013; Thyme et al., 2014). This is because the 3D structure of the protein and amino acid composition of its active centre are responsible for the HEG specificity. The HEG’s cleavage and target recognition functions are located within the same regions of the protein, so modifying sequences to confer binding specificity may compromise endonuclease efficiency. Also, the amino acids responsible for the formation of base pair-specific contacts are not modular and may function in a context-dependent manner. To switch target sites means that the HEG must be re-engineered at the DNA level and assessed through a screening process to identify the optimal structure that recognises the new target site. Several gene editing technologies have been used to replace native HEGs with more easily programmable nucleases. Zinc finger nucleases (ZFN) (Kim et al., 1996; Urnov et al., 2010) and transcription activator-like effector nucleases (TALENs) (Miller et al., 2011) are simpler to engineer than HEGs and have been tested for their functionality in the mosquito (Smidler et al., 2013) and for their efficiency under a gene drive scenario (Simoni et al., 2014).

The discovery of the CRISPR/Cas9 system has revolutionised the field of gene drives and has been rapidly adopted by the scientific community, replacing almost all other genetic engineering tools previously used to alter the genome of mosquitoes (as well as other organisms). The simplicity of the system and ease of engineering has set it apart from earlier gene drive technologies using HEGs, ZFN or TALEN nucleases. The most used variation of the CRISPR/Cas9 system is derived from *Streptococcus pyogenes* (Jinek et al., 2012) and has been immediately used as an alternative tool in homing-based gene drive systems. Proof-of-principle experiments have been performed in yeast (DiCarlo et al., 2013) and fruit fly (Gantz and Bier, 2015), before the system was adapted in gene drives for mosquito population replacement and suppression in mosquito populations kept in ACL2 containment laboratories. In 2015, Gantz et al. (2015), used the CRISPR/Cas9 system to bias the inheritance of anti-malaria effectors in the progeny of hemizygous *Anopheles stephensi* mosquitoes. The gene drive could home in the germline of the mosquito with up to 99% efficiency, though a cage-trial was not performed in that study (Gantz et al., 2015).

Around the same time, the CRISPR/Cas9 system was utilised by researchers at Imperial College London to disrupt three female fertility genes in *Anopheles gambiae* (Hammond et al., 2016). Two *Drosophila melanogaster* orthologues *yellow-g,* and *nudel*, (AGAP005958 and AGAP007280 respectively), and AGAP011377, which has no apparent *Drosophila* orthologue, were targeted with a synthetic CRISPR/Cas9-based system to study the effects on female reproduction, with the aim of suppressing the mosquito population. One of those gene drives, targeting the gene *AGAP007280,* was released in caged mosquito populations to study the dynamics of the system at the locus. The gene drive was released at a 50% transgenic frequency using hemizygous gene drive mosquitoes. As expected from model predictions, the gene drive increased in frequency within 6 generations, reaching 75–80% transgenic ratio (Hammond et al., 2017). Due to the selective pressure the gene drive posed for resistant alleles that were created by the nuclease activity on the *AGAP007280* locus, mutations at the target site that restored the function of the *AGAP007280* gene had a selective advantage over the gene drive that was deliberately designed to impose a fitness load on the population. These target site mutations with restored *AGAP007280* gene function gradually increased in frequency in the caged populations, counteracting the function and spread of the gene drive. The creation and selection of resistant alleles is a major issue for gene drive technology that must be considered when designing such systems, especially those destined to impose a selective pressure on a population.

A strategy to mitigate the occurrence of the resistance to a gene drive is to target regions that are functionally and structurally constrained. The effectiveness of this approach was demonstrated by targeting the female-specific isoform of the sex determination gene *doublesex* (*dsx*) in *A. gambiae* (Kyrou et al., 2018). By disrupting the female-specific isoform, *dsxF*, individuals homozygous for the *dsxF* knockout were intersex and unable to blood-feed, so did not produce any eggs. Meanwhile the males carrying the null mutation in *dsxF* were unaffected, both in hemizygosity (*dsxF* ^+/−^) and homozygosity (*dsxF* ^−/−^) (Kyrou et al., 2018). Compared to the gene drive at the *AGAP007280* locus, when targeting *dsx*, the gene drive was able to spread into a caged mosquito population reaching a frequency of 100% within 7–11 generations causing a population collapse. At the target site there was a very low frequency of indels (up to 1.16% frequency among non-drive alleles) with no evidence that any of them were positively selected (Kyrou et al., 2018).

## 2 Lessons learnt towards the next generation of gene drives

One crucial reason why the CRISPR/Cas9 homing gene drive system targeting *AGAP007280* was prone to fail is the low conservation that characterised the target sequence of the gene, resulting in the development and selection of gene drive resistance at the target loci (Hammond et al., 2017). A high level of variation implies that a locus is not functionally constrained and most probably can tolerate in-frame mutations, like the ones observed in the cage population experiment (Hammond et al., 2017).

The increasing genomic resources available have allowed conservation analysis to be performed across the *Anopheles* species complex to identify regions that are highly conserved (Kranjc et al., 2021; O’Loughlin et al., 2021). The use of these bioinformatic resources can help guide where to target a gene drive, such that it is at a genomic site that could prove intolerant of mutations. Furthermore, the *Anopheles* 1,000 Genomes Consortium provides whole genome sequencing data on the genetic variation in wild populations of *A. gambiae.* With the phase 3 SNP data release (Ag3) this now encompasses 2,874 wild *Anopheles* mosquitoes that have been sampled across Africa (The Anopheles gambiae 1000 Genomes Consortium, 2021). Therefore, the choice of target site can now combine both conservation across the *Anopheles* species complex, and the genetic variation of field-sampled *Anopheles* mosquitoes to find sites that display high levels of conservation. This reduces the likelihood of resistance occurring to the drive because of the standing genetic variation, also these could be sites that are functionally constrained. However, research has shown that resistance can occur at such an evolutionary conserved site (Fuchs et al., 2021), and that genomic sites can stay ultra-conserved, even without apparent functional constraint (Díaz-Castillo et al., 2012), so might be able to endure mutations caused by a CRISPR/Cas9 gene drive. Notably, the *dsx* gene drive targets a conserved site at an intron-exon boundary which is critical for its correct pre-mRNA splicing. Fuchs *et al* hypothesised that sequences important for RNA processing (such as the sequence targeted in the *dsx* gene drive) may have less degeneracy, indicating that targeting sequences that lie in non-degenerate codons, or the non-wobble positions of degenerate codons, may add additional levels of resilience to a gene drive (Fuchs et al., 2021).

The mutations that arise from CRISPR/Cas9 editing are caused by the DNA being repaired by non-homologous end-joining (NHEJ), rather than HDR where the CRISPR construct should be copied over. Therefore, to reduce the number of mutations being generated, it is desirable to bias the DNA repair towards HDR, minimising NHEJ. The *vas2* promoter that was used to drive expression of Cas9 in the original system targeting *AGAP007280*, is known not to be tightly restricted to germline expression, and causes maternal deposition of Cas9 into the embryo (Papathanos et al., 2009; Hammond et al., 2021, 2017). This can then cause substantial end-joining mutations in the germline, that could become resistance alleles to the gene drive. Furthermore, somatic editing by Cas9 was observed that had a strong fitness cost in females hemizygous for the drive; there was conversion to homozygosity for the null allele due to mutations arising in the soma (Hammond et al., 2021). To negate this effect, different germline promoters were tested for Cas9 expression to improve the temporal restriction to the germline, and so reduce the propensity for end-joining mutations to occur. Of these, the *AGAP006241* (*zero population growth*, *zpg*) promoter showed the best improvement for use in a gene drive setup. High homing was maintained (>90% in males and >97% in females), but a great reduction in end-joining mutations and parental deposition was observed for the *AGAP007280* locus, whilst females hemizygous for the drive had a reduced fecundity cost (Hammond et al., 2021). However, the *zpg* promoter did show some evidence of paternal (Kyrou et al., 2018) (*dsx locus*: female fertility) and maternal effects (Fuchs et al., 2021) (*AGAP029113* locus: viability) showing the size of the parental effect could vary depending on locus. However, when this promoter was incorporated into the previously described gene drive targeting *dsx* it was able to cause population collapse, indicating that the parental effect is below the threshold it would take to inhibit the drive (Kyrou et al., 2018).

A further measure to reduce the impact of resistant alleles being created at the target site in future gene drive systems is to multiplex gRNAs targeting various loci in proximity (Port and Bullock, 2017; Champer et al., 2018, 2020; Oberhofer et al., 2019). Multiplexing gRNAs like this ensures that even if resistant alleles are formed in some of the targeted loci, they will not impair the homing process. This is because in theory only one gRNA, *i.e.,* one double strand break, is needed for the homing process to occur. The idea behind the use of multiple gRNAs ensures that a gene drive system has enough time to invade the population and cause a significant reduction before all the target sites confer resistance. In *Anopheles,* gene drive systems that use two gRNAs to target *dsx* are currently under development and will soon be ready to be tested in small cage experiments.

## 3 Endogenous gene targets for more efficient CRISPR-based homing drives

One crucial component for the development of efficient gene drive elements that use the CRISPR/Cas9 homing system is the identification of appropriate endogenous gene targets. Gene targets with functional or structural constraints are less likely to tolerate mutations or contain nucleotide polymorphisms in the target populations, even though extensive genome variability exists in mosquito populations.

Advances in RNA sequencing technologies, particularly third generation sequencing—*i.e.,* long-read sequencing that facilitate isoform-length RNA sequencing (Pollard et al., 2018)—coupled with the substantial improvements of the genome assemblies for the available species of the *Anopheles gambiae* complex (Neafsey et al., 2021)—have led to significant improvements in the identification of functional genes.

Homing-based drive systems could be used for population replacement strategies to either introduce novel genes that enhance the mosquitoes’ immunity against malaria infection, or to disrupt endogenous genes that are essential for the survival/development of the malaria parasite in the mosquito vector.

For self-sustaining homing-based drive systems, targeting endogenous genes that are essential for the survival or fitness of the malaria mosquito may also be used for population suppression (Burt, 2003, 2014). If a mosquito population is reduced significantly in size so that it can no longer support the malaria parasite population, disease could be eliminated.

**FIGURE 1:**
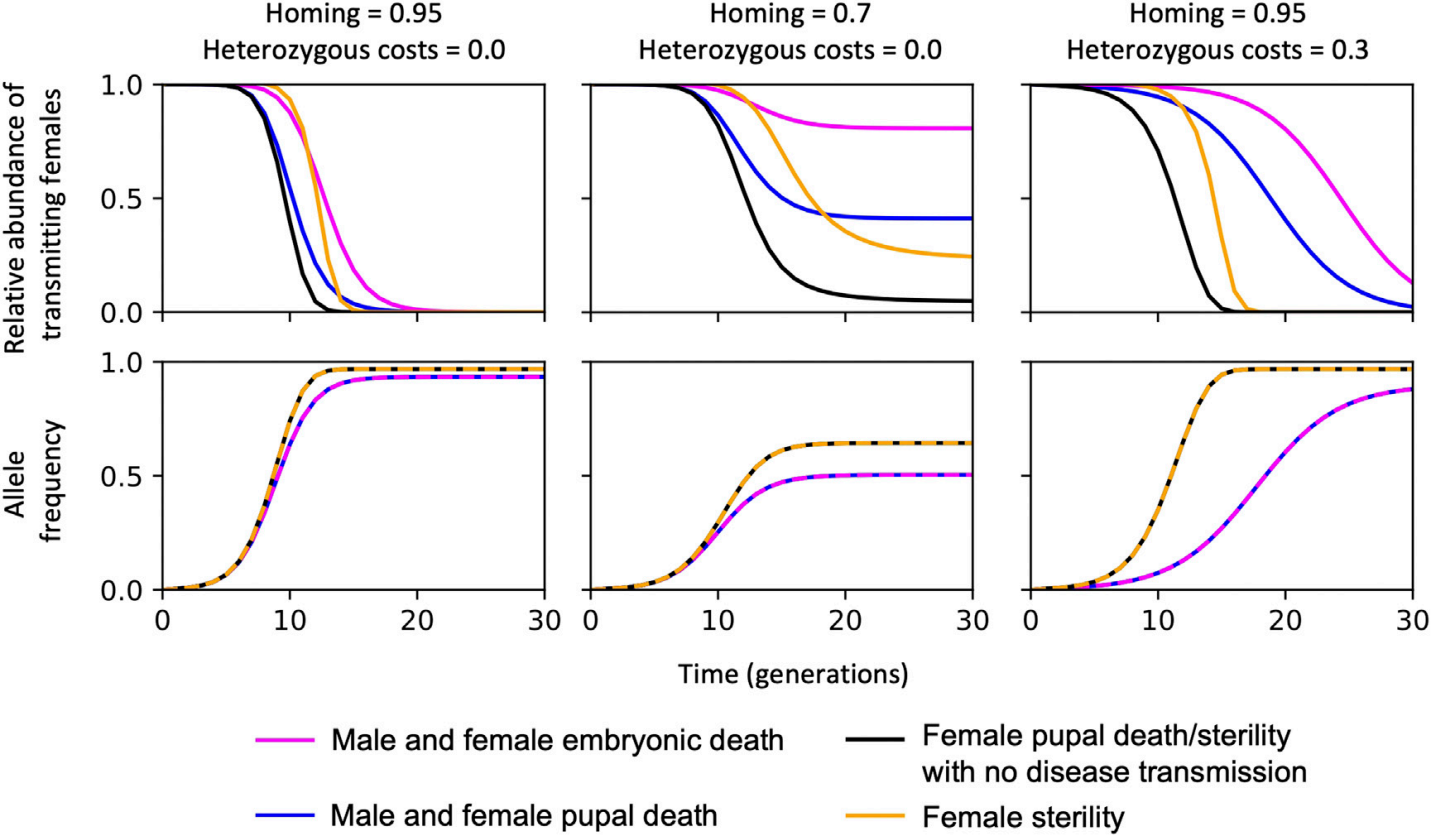
The efficiency of self-sustaining CRISPR-based suppression gene drive systems with different gene targets to reduce disease-transmitting mosquitoes. In each case, the CRISPR-gene drive construct is inserted into a haplo-sufficient site, assuming homozygotes are either completely inviable with death occurring: (i) at the embryonic stage (“Male and female embryonic death”); (ii) as pupae, or at the pupal-adult eclosion boundary, i.e., no adults emerge (“Male and female pupal death”); (iii) in just the females as pupae/pupal-adult eclosion boundary, equivalent to all females surviving but having no ability to transmit malaria or reproduce “female pupal death/sterility with no disease transmission”; or “female sterility” with no deaths occurring such that females can still transmit malaria but cannot reproduce. Summary of findings reported in (Deredec et al., 2011; Burt and Deredec, 2018) using a non-spatial model and parameterisation from (Burt and Deredec, 2018; Kyrou et al., 2018; Hammond et al., 2021).

Although a range of mosquito life cycle stages could be targeted by gene drive constructs—such as genes that are essential for the development of embryos, pupae, or adults—the efficiency of the self-sustaining gene drive is affected by strong density dependence effects in *An. gambiae* larvae, which impact their survival and development (Burt and Deredec, 2018). The effect of density dependence on the gene drive efficiency is summarised in Figure 1; a self-sustaining homing construct targeting a gene essential for mosquito development is more efficient at removing the disease-transmitting female population if death occurs after density-dependent mortality.

Also (summarised in Figure 1), since mosquito-borne diseases are exclusively transmitted by the female mosquitoes, genes implicated in female-specific functions present more desirable targets (Deredec et al., 2011; Burt and Deredec, 2018; Kyrou et al., 2018; Hammond et al., 2021). Importantly, the allele frequency of the self-sustaining construct increases more efficiently when the gene target is female-specific since fewer construct alleles are lost per generation because the transgenic male population are unaffected (Figure 1).

Finally, the most efficient construct for removal of disease transmitting females—*i.e.,* showing efficient reduction in the relative abundance of disease transmitting females relative to the starting population over time (Figure 1)—are those causing female-specific pupae/adult deaths or where the adult female is unable to reproduce and transmit disease. This is because there would be minimal effect on larval density, males are unaffected and transgenic homozygote female adults are unable to transmit. There are also subtle differences when reducing the homing rates and hemizygous fitness costs, however the constructs targeting female pupal death/sterility has a robust pattern and performs best, Figure 1 (middle and right panels).

Though no gene target has so far been identified in *An. gambiae* for a gene drive construct that converts genetic females into phenotypic males, such a construct would be expected to be even more efficient since there would be no construct alleles lost per generation, density-dependence mortality would not be affected and a reduction in disease-transmitting mosquitoes would occur at the same rate as if the construct was female-lethal (or sterile and unable to transmit disease).

There are also more complex dynamics of refugia, extinction and recolonisation that are considered in spatial models that could impact the efficiency of the gene drive scenarios shown in Figure 1 (Eckhoff et al., 2017). For example, spatial modelling of the suppression of *An. gambiae* using the CRISPR/Cas9 gene drive targeting *dsx* to reduce female fertility predicts less suppression than equivalent non-spatial models, particularly in more seasonal regions where the mosquitoes need to survive the dry season (North et al., 2020). It would be useful to explore and compare the interaction between the gene drive scenarios in Figure 1 (with their different resulting phenotypes, *i.e*., bi-sex/sex-specific affects on fertility/survival) and climatic and environmental variables to determine the desirable strategy that best meets the population suppression strategy in the target area of high malaria prevalence that may be considered for genetic vector control. Potential gene targets that are expected to affect female *Anopheles* death/sterility and the inability to transmit disease are discussed below.

### 3.1 Candidate endogenous gene targets

#### 3.1.1 Mosquito flight

Is important for mosquito survival and reproduction, and some genes involved in mosquito flight are female-specific, making them attractive targets for population suppression. Mating takes place in flight, wingbeat frequency is an important sexual recognition clue (Wheeler, 1962; Cator et al., 2009; Gibson et al., 2010), and females rely on flight to reach their blood meal source, necessary for eggs production. Indirect flight muscles (IFMs) located in the adult thorax of mosquitoes provide the mechanical power that support flying (Lawrence, 1982).

The *Actin-4* gene involved in female mosquito flight was identified and characterised in *Aedes aegypti*—the mosquito vector of dengue, chikungunya, and Zika*—*and *Culex quinquefasciatus*—the mosquito vector of human parasitic worm (*Wuchereria bancrofti*) and the West Nile virus (Muñoz et al., 2004; Labbé et al., 2012; Navarro-Payá et al., 2020). Actin-4 is a specific actin isoform that polymerases to form the actin filaments in the IFMs. This role is played by the paralog gene, *Act88F*, in *D. melanogaster*, where, unlike in mosquitoes, this gene is expressed in both males and females (Lawrence, 1982; Hiromi and Hotta, 1985; Okamoto et al., 1986). Dominant female-flightless *Actin-4* mutants were identified in both *Ae. aegypti* and *C. quinquefasciatus* highlighting the potential for this gene in vector control strategies (Navarro-Payá et al., 2020). In *Ae. aegypti,* knockouts of *AeAct-4,* as well as *myo-fem,* a sex-biased myosin gene, led to 100% female flightless; males were able to fly and were only slightly less competitive than the wild type, suggesting involvement of this gene in mating success. A phylogenetic analysis of actin protein sequences across multiple mosquito species identified orthologous of these genes in *Ae. albopictus*, *Culex*, and *Anopheles* mosquitoes (O’leary et al., 2020).

#### 3.1.2 Sex determination

The sex determination pathway in insects contains some of the most conserved genes in the class and as such are potentially excellent targets for a population suppression strategy. Previously, we discussed the effectiveness of a gene drive in *A. gambiae* that targets *dsx* (Kyrou et al., 2018), but other elements of the sex determination have been identified in *A. gambiae*: *fruitless* (*fru*), *femaleless* (*fle*) and *Yob*. The splicing of *dsx*/*fru* into their respective productive and non-productive forms in the female *A. gambiae* was shown to be controlled by the upstream element, Fle (Krzywinska et al., 2021). Knockdown of *fle* was able to cause female masculinisation and death (depending on level of knockdown), which given the levels of knockdown seen, indicated that *fle* is a haplo-insufficient gene (in females), such that it would not be a suitable target for a self-sustaining gene drive. It is not currently known whether *fle* needs a co-factor, as for *D. melanogaster* where a Tra/Tra2 is needed for the splicing of *dsx*/*fru* (Inoue et al., 1992), however so far, the search for a *tra* orthologue in *An. gambiae* has not yielded a candidate gene. If identified in *An. gambiae,* a Tra/Tra2 orthologue—the primary instructive signal of the sex determination cascade being the binary genetic switch gene *Tra* and its cofactor, Tra2—could provide a more efficient gene drive target for homing gene drives if the null homozygous allele would result in female to male sex conversion (Karaminejadranjbar et al., 2018; Aumann et al., 2020), with no effect on dosage compensation.

Along with *dsx*, *fru* is alternatively spliced into separate female (*FruF*) and male (*FruM*) isoforms in *A. gambiae* in the sex determination pathway. In *D. melanogaster* the females do not produce a functional *fru* protein however in males a functional protein is produced that is involved in the regulation of male sexual behaviour (Ryner et al., 1996; Gailey et al., 2006). The pattern of *fru* production in *An. gambiae* is very similar to that of *D. melanogaster*, displaying high conservation (Gailey et al., 2006; Krzywinska et al., 2021). Unlike the Tra/Tra2 complex that causes sex-specific splicing in *D. melanogaster* females, for *An. gambiae* it was shown that Fle is involved in the sex-specific splicing of *fru*. When *fle* was knocked down in *An. gambiae* females, they produced the male version of *fru* and were not attracted to blood (Krzywinska et al., 2021), indicating that it could be possible to affect the fertility of females by targeting *Fru* ^F^.

#### 3.1.3 Blood feeding and digestion, and midgut immunity

As blood feeding is a behaviour exclusive to the female mosquito, and the site of blood digestion–the midgut–is also the site of malaria parasite development, genes involved in this process are of particular interest for both suppression and replacement strategies. Specifically, genes expressed in the female midgut post-blood meal (PBM) are commonly required for digestion and immunity; this is reflected by the increased complexity in gene expression of female midguts compared to males (Warr et al., 2007). Previously, the upstream regulatory regions of midgut-specific and/or blood-induced genes have been used as putative promoters to drive expression of transgenes *in vivo* (Edwards et al., 1997; Nolan et al., 2011; Miglani et al., 2017; Haghighat-Khah et al., 2019). This has proven extremely useful to better analyse parasite movement in the midgut as well as for targeted delivery of anti-parasitic agents (Isaacs et al., 2012; Volohonsky et al., 2020).

For use in population suppression, gene drives targeting genes expressed PBM could function as inducible effectors since any effects on the fitness of females would occur after a blood meal. This would also allow transgenic females to develop normally and compete with wild populations for space, resources and mating opportunities as an alternative to existing strategies. Genes involved in blood digestion and immunity in the midgut could be promising targets for such a drive. For example, knockout mutants of fibrinogen-related protein 1 (*FREP1*), a gene known to facilitate plasmodium invasion in the midgut, displayed significant resistance to infection by the malaria parasite and severe fitness costs (Dong et al., 2018). More recently, midgut promoters with increased expression PBM were used to target parasite development in a population replacement gene drive (Hoermann et al., 2021).

Several genes with induced midgut expression PBM have been identified in *An. gambiae* that could be used as new targets for gene drive (Cui et al., 2020). Examples include members of the trypsin gene family shown to be essential in blood digestion that have midgut-specific PBM-induced expression (Müller et al., 1993; Muller et al., 1995), although there are many digestive enzymes in the midgut and so targeting multiple enzymes simultaneously (such as using multiple guide RNAs) may produce a more suitable phenotype. Other potential gene targets include those involved in midgut immunity such as the *Anopheles* heme peroxidase HPX15, which has been shown to supress the immune response to the malaria parasite in *An. stephensi;* this was observed by a drastic decrease in the number of developing oocysts forming following RNA interference-mediated silencing (Kajla et al., 2017) A number of blood-induced midgut genes have also been identified in *An. gambiae* that have a direct effect on transmission of *Plasmodium falciparum* (Cui et al., 2020).

### 3.2 Fitness costs

Fitness costs induced by engineered gene drive elements play an important role in the efficiency (and therefore utility) of the gene drive. An early example of the significance of non-target fitness costs was demonstrated in the 1970s when Curtis suggested using translocation homozygotes to drive refractory genes into the targeted wild mosquito populations (Curtis, 1968), which was attempted for the first time in a field trial in Kenya (Lorimer et al., 1976). The project failed due to severe fitness deficits of the released strain, which was not a transgenic strain. If there is a fitness cost associated with a transgenic gene drive element, the release ratio required for fixation increases substantially (Gould et al., 2008; Akbari et al., 2013), though in these circumstances, release over multiple generations permits a lower release ratio to achieve fixation, though with a high number of released insects overall (Magori and Gould, 2006; Akbari et al., 2013).

Non-target fitness costs in homing-based gene drives may be associated with the presence of the transgenic constructs due to leaky expression of transgene(s) (*i.e*., expression of transgene(s) outside of the intended temporal and/or spatial range that was originally designed) that may prevent their correct functioning and regulation (Esnault et al., 2011). These effects may be attributed to heterochromatin (Wallrath and Elgin, 1995) and enhancers or silencing elements in the DNA flanking the transgenes. Unpredictable disruptions may also induce a fitness penalty if the targeted endogenous gene is involved in additional unidentified essential functions, or due to interference with coding or regulatory sequences close to the integration site, or regulatory elements within the construct that may impact expression of endogenous genes in *cis* and *trans* (Wilson et al., 1990; Mellert and Truman, 2012).

In addition, for homing-based drives, which require a targeted double strand break to induce the cell’s homology-directed repair machinery, it is also important to assess and manage the potential risk associated with cleavage at off-target sites, *i.e*., genomic mutations occurring at an unintentional loci that has a similar (or identical) sequence to the target site. The number of off-target effects is less overall following genome editing techniques compared to randomly induced mutations (European Commission et al., 2019), such as due to irradiation used in conventional SIT vector control. It is also challenging to distinguish between spontaneously occurring mutations (in the absence of any induced mutations), and off-target mutations potentially induced by site-directed nucleases (such as CRISPR/Cas9). Most off-target mutations would be expected to have no phenotypic effect, and so be of no consequence, whereas others may reduce the fitness of the mosquito (or affect epidemiologically important traits such as pathogen susceptibility or resistance to insecticides), and collectively at the population level, their accumulation may influence their outcome (James et al., 2020). An *in vivo* study of off-target mutations for CRISPR-Cas9 homing gene drives including one with a deliberately promiscuous set-up (with a gRNA targeting a sequence with many closely related sites of ≥2 mismatches and a non-germline-restricted promoter for the nuclease) demonstrated that although some off-target mutations were observed (>1.42%), there was no evidence that these accumulate, or drive, in the mosquito population, despite multi-generation exposure to the CRISPR/Cas9 construct (Garrood et al., 2021). While this lab study demonstrated that CRISPR/Cas9 off-target editing can be reduced to minimal levels in *An. gambiae* gene drives through the use of bioinformatics and *in vitro* testing (Garrood et al., 2021), an important consideration noted by the authors is the genetic diversity that a gene drive would face in the wild, with polymorphisms that could potentially result in the presence of genomic target sites that closely resemble the target site. Another practical way to evaluate off-target effects is by assessing phenotypic changes (such as fitness), since the heterogeneity would complicate genome sequencing analysis (James et al., 2020).

For the population replacement drives, engineered malaria refractoriness may also impose a fitness penalty on the insect due to an evolutionary cost of mounting an immune response (Koella, 2003). This may lead to silencing of the introduced refractory transgenes in response to a strong fitness load caused by the genomic integration (Adelman et al., 2004), whilst targeting a neutral genomic site that shows poor conservation induces the evolution of resistance to the gene drive element (Beaghton et al., 2017; Noble et al., 2017).

Recently, the use of marker-less and promoter-less gene drive elements inserted as terminal fusions of endogenous germline and somatic genes (known as “integral gene drives”, IGDs; Supplementary Table S1) was proposed to reduce negative fitness consequences (Nash et al., 2018). A proof of principle experiment in *An. gambiae* showed the successful engineering of marker-less and promoter-less genetic modifications using the CRISPR/Cas9 homology-based recombination followed by a separate Cre-*loxP* excision. The removal of extraneous transgenic sequences could also be performed using FLP-*FRT* excision, as demonstrated in the integrase-recombinase mediated cassette exchange (iRMCE) gene editing system in *Aedes* mosquitoes (Haghighat-Khah et al., 2015).

Analysis of the IGD elements indicated that modifications of the targeted midgut-specific host genes effected infection outcomes in various ways, either reducing or increasing malaria transmission (Hoermann et al., 2021). This highlights the variability of possible outcomes that are synonymous with positional insertion effects, though the development of marker-less and minimal gene drive elements presents an opportunity to reduce extraneous transgenic sequences and their effects for future deployment of the gene drive technology. However, it is important to consider that the presence of unique, visible or molecular markers is required for the identification of transgenic arthropods for containment purposes (ACME ASTMH, 2022) and monitoring efforts (WHO, 2021b). Whilst detection of molecular markers using large-scale gene amplification technologies could be feasible (WHO, 2021b), these are expensive and challenging for colony maintenance and the absence of visible markers could pose a significant risk to other mosquito colonies kept in the same insectary.

### 3.3 Anti-gene drive systems and approaches to restrict impacts of gene drives to a target population

Prior to any release of a gene drive system, rigorous risk assessment is expected to ensure that all potential risks are identified, evaluated and mitigated. In addition to this, several possible systems and approaches have been proposed to control the spread of a gene drive element following their release. As with any other strain of genetically-modified mosquito, an anti-drive strain (or any other lab-developed strain intended for field release) would also require rigorous testing, risk assessments and regulatory approval prior to use in the field. Research into these strategies is at an early stage, and the level of success (*i.e*., its ability to reverse a gene drive in the lab or in the wild) is currently difficult or even impossible to predict (Geneva: World Health Organization, 2021; National Academies of Sciences, Engineering, and Medicine, 2018; van der Vlugt et al., 2018).

Strategies to allow the spread of a gene drive within targeted populations but preventing their spread to non-target organisms includes targeting unique species-specific sequences (Australian Academy of Science, 2017; Geneva: World Health Organization, 2021; German Central Committee on Biological Safety, 2016; NASEM, 2016; van der Vlugt et al., 2018; World Health Organization, 2016). A diverse group of *Anopheles* (>30) species transmit human malaria, and of these, the *An. gambiae* species complex contains the most important malaria vectors in sub-Saharan Africa (O’Loughlin, 2020). Although some studies have shown that lab colonies of different species can cross-mate and produce viable eggs (Liang et al., 2021), five species do not overlap geographically, whilst there are pre-mating barriers between other species (Diabaté et al., 2009). Notably though, hybridisation has been observed between wild *An. gambiae* and *An. coluzzii*, *e.g.*, ranging from 0.07 to 1.29% in three western Burkina Faso villages (Epopa et al., 2019) to ∼21–33% in a coastal village in Côte D’Ivoire (Caputo et al., 2022), whilst some rare wild hybrids (<0.1%) have been detected between other sympatric species (O’Loughlin, 2020). With the expanse of population genomic data in more species and locations, the identification of highly conserved target sequences that are unique to a target species and its geographic region (“private alleles”) would facilitate gene drive designs that reduce the risk of spreading beyond the target population (Willis and Burt, 2021). The detection of PAM sequences—protospacer adjacent motif: 2-6 bp sequence immediately following the target sequence that is essential for the correct binding and cleavage of DNA by the CRISPR/Cas9 system—that are completely private to *An. gambiae* populations in a targeted geographic range, indicates the feasibility of restricting homing gene drives to target mosquito populations (Willis and Burt, 2021).

Approaches to limit the impact of a gene drive intervention to a target population includes the use of self-limiting drives that are transient due to a non-driving helper construct, such as killer-rescue systems (Gould et al., 2008), split drives (Oberhofer et al., 2021; Terradas et al., 2021) and “daisy chain” drives (Noble et al., 2019), see Supplementary Material. Other approaches include the use of high-threshold gene drives that are lost from the population if the threshold frequency is not exceeded, such as underdominance strategies where hemizygotes are less fit than the dominant or recessive homozygotes (Davis et al., 2001; Champer et al., 2021), tethered drives (Dhole et al., 2019) or split drive killer-rescue systems (Edgington et al., 2020). These high release threshold approaches require the production and release of a larger number of gene drive mosquitoes than self-sustaining gene drives, whilst achieving the balance between ability to spread and remain locally confined is challenging (Dhole et al., 2020).

Further alternatives or additional approaches would be creating organisms or variants with genome editors susceptible to a lack of certain nutrients or to a presence or lack of certain chemicals in the environment, such as Tet-On/Tet-Off or other small molecule systems. This approach aims at inhibiting or blocking genome editing activity in an organism (López Del Amo et al., 2020). Monitoring levels of chemicals/nutrients in the target populations’ breeding sites and surrounding areas is crucial for the success of the strategy. For example, the absence of tetracyclines in the wild is required for a Tet-Off system to work, so tetracycline contamination must be evaluated in the breeding sites and surrounding areas of the targeted mosquito species. As part of ongoing risk assessments evaluating the use of a non-driving self-limiting genetically modified strain (OX513A) for the control of dengue fever, the concentration of tetracyclines and its analogues detected in known *Ae. aegypti* breeding sites and surrounding areas was lower than the minimum required concentration shown to inhibit a Tet-on/off system (Curtis et al., 2015). To limit the spread of conditional expression systems, if the tTA was replaced with reverse tTA in a Tet-On system, the gene drive would only spread in the presence of tetracycline in breeding sites, thus limiting its spread such that a gene drive system could be evaluated somewhat before release of a gene drive lacking the conditional component (Urlinger et al., 2000; Scott et al., 2018).

Alternatively, reverse genome editing to restore a previously edited genome to its initial state has also been proposed (HCB Scientific Committee, 2017; National Academies of Sciences, Engineering, and Medicine, 2018). These may simply include the release of the recipient organism either:

• in its unaltered state, though this will still be susceptible to the gene drive element if complete elimination was not achieved beforehand because the gene drive’s target sequences is intact; or
• carrying pre-existing alleles that are not receptive to gene drives (*i.e*., are refractory to cleavage due to the absence of the target site: “drive-refractory alleles”);
• carrying alleles that are not receptive to gene drives that were generated through NHEJ repair of endonuclease-induced cleavage, as long as these alleles do not have impaired fitness.

Genetically modified organisms carrying a second gene drive element could be used to restore the wild type phenotype (Bundy and Fanning, 2005; Mumby and Steneck, 2008; Akbari et al., 2015; National Academies of Sciences, Engineering, and Medicine, 2018; van der Vlugt et al., 2018). Notably, if the gene drive reversal system is targeting the original gene drive, for example using a CRISPR/Cas9 system designed to target a transgenic sequence, once there are no more transgenic sequences available in wild populations for the gene drive to target, the gene drive reversal system and all its transgenes will be lost from the population due to Mendelian inheritance (Australian Academy of Science, 2017; Geneva: World Health Organization, 2021; German Central Committee on Biological Safety, 2016; HCB Scientific Committee, 2017; NASEM, 2016; van der Vlugt et al., 2018; World Health Organization, 2016).

Recently, the transgenic expression of the anti-CRISPR protein AcrIIA4—a *Listeria monocytogenes* prophage protein—in *An. gambiae* inhibited the super-Mendelian inheritance of the gene drive element until both anti-drive and gene drive elements reached a state of equilibrium, preventing the suppression and collapse of caged population, whereas the gene drive-only control cages crashed following 6 generations as expected (Taxiarchi et al., 2021). The gene drive inhibition in this system is *via* protein-protein interaction, which circumvents potential unintended effects due to alternative Cas9-gRNA or gRNA-only reversal systems that further modify the mosquito genome in sometimes unpredictable ways (Esvelt et al., 2014; Gantz and Bier, 2016; Wu et al., 2016; Xu et al., 2020). Notably however, the anti-CRISPR protein AcrIIA4 acts indiscriminately by interacting with and inhibiting the Cas9-gRNA complex, such that it would interact and block the super-Mendelian inheritance of any future released CRISPR/Cas9-based gene drive element as well, thus limiting its utility in the field.

The gene drive countering approaches discussed here are still undergoing further development. Current research is concerned with, amongst other things, reducing evolution of resistance to gene drive, developing a gene drive mechanism whose spread would be limited, and designing tools able to reverse an existing gene drive (NASEM, 2016; HCB Scientific Committee, 2017). These approaches collectively contribute to the ongoing efforts to evaluate any potential risks associated with the release of any gene drive system. Moreover, unintended effects following the release of a gene drive may theoretically occur before a mitigation strategy takes effect (Geneva: World Health Organization, 2021; van der Vlugt et al., 2018). These impacts could be reversed (Shapiro and Wright, 1984) for example, by re-introduction of the eliminated wild type species (Bundy and Fanning, 2005; Mumby and Steneck, 2008; NASEM, 2016).

As for any driving or non-driving genetic control systems intended for field release, the utilisation of any of the discussed gene drive reversal strategies will require complete assessment and authorisation for the release of genetically modified organisms according to national or international regulations, e.g., Directive 2001/18/EC19 (Geneva: World Health Organization, 2021; van der Vlugt et al., 2018).

### 3.4 A close match to wild mosquitoes

The success of gene drive mosquitoes that are expected to have a multigenerational effect relies on measures of competitiveness, longevity, and the duration of the population effect to understand the scale of releases that are needed to achieve the intended outcome. These measures are significant for strain efficacy evaluation and estimations of delivery requirements (*e.g*., release ratios and number/duration of releases). Any loss of fitness and reduction in the frequency of the gene drive allele must be balanced by the greater-than-Mendelian inheritance rates (>50%). Modelling can be used to refine efficacy predictions based on the results of small- and large-cage testing (Wu et al., 2021). Thus, assessing fitness parameters, specifically the ability of gene drive mosquitoes to survive, mate and reproduce, is of great practical importance, and also forms an essential component of the risk assessment prior to any release.

Inbreeding and harmful effects of homozygous recessive genes have been suggested as causes for reduced fitness in transgenic field releases (Marrelli et al., 2006). However, making transgenic strains using highly inbred lab colonies is not always deleterious (Allen et al., 2004; Moreira et al., 2004). For example, lab colonies could be refreshed by breeding with several geographically distinct populations, known as a genetically diverse laboratory strain or GDLS (Wise de Valdez et al., 2010).

The offspring of a gene drive mosquito that has mated with a wild mosquito in the field will also carry the other (non-driving) genes of the gene drive parent that enter the populations gene pool, though they will be inherited at the Mendelian inheritance rate (50%). If there is any associated fitness cost, non-driving genes would be inherited less than the Mendelian inheritance rate, which would result in their decline in frequency over time. This was demonstrated when genomic sequence variants (single nucleotide polymorphisms; SNPS) of the transgenic *Ae. aegypti* OX513A colony, which carries a non-driving self-limiting transgene with >5% survival of offspring (Phuc et al., 2007), were detected in field caught mosquitoes (“hybrids”) after release commenced, indicating that a few of the survivors could reproduce, though their numbers rapidly declined post-release (Evans et al., 2019, 2020). Gene flow from non-driving genomic sequences of released strains is unlikely to have an impact (Alphey, 2019), unless introgressed genes result in, for example, either insecticide resistance or increased vector competence, which are factors that would be identified and thoroughly evaluated directly in release strains prior to any potential field release (Connolly et al., 2021). Notably, a derivative strain of *Ae. aegypti*-wMel—a non-genetically modified gene drive system that exploits the maternal inheritance of the reproductive parasite *Wolbachia* in *Ae. aegypti* mosquitoes to bias the inheritance of Dengue-resistance in mosquitoes—that carry more insecticide resistance alleles (*w*MelRio) was intentionally developed to match the wild target mosquito population, and were released to establish the gene drive mosquitoes in the field (Garcia et al., 2019). This work highlights the importance of matching local population genetics to achieve the goal of the released gene drive system to spread desirable traits to the target population.

The degree of introgression of non-driving sequences from released strains could be reduced (but not eliminated) by backcrossing of the gene drive strain to a local wild type strain for multiple generations before release, or the gene drive element could be inserted into local mosquito strains per field site. This may improve mating between released gene drive mosquitoes and the target population in the wild, however more wild-like mosquitoes will be less lab-adapted and therefore likely to be difficult to rear and more costly to produce.

### 3.5 From scientific discovery to application

Although some engineered gene drive organisms have been developed and tested in the lab, to date, none have been studied in the open environment. The question of whether and how an engineered gene drive will be considered ready for intended field release has generated considerable discussion, with a general consensus on the significance of incremental phased testing pathways, which increase the level of human and environmental exposure to engineered gene drive mosquitoes in a step-by-step way (WHO, 2014; NASEM, 2016; James et al., 2018). A modified phased testing pathway was recently published as guidance for testing self-sustaining (such as homing-based) gene drives to incorporate additional safety considerations due to their persistence and spread, which includes the following updates (WHO, 2021b):

• when Phase 1 (confined lab studies or studies in indoor population cages/environmental chambers) are conducted in regions hospitable to the targeted species, additional precautions are necessary to avoid escape into environment.
• for self-sustaining, non-localizing gene drive modified mosquitoes, testing in a series of discrete phases may not be possible after Phase 1, but field testing may better be conceived of as a continuum of expanding releases. Therefore, safety testing in Phase 1 and thorough risk assessment prior to Phase 2 (physically and/or ecologically confined field studies; “semi-field”) will be especially important for these gene drive modified mosquitoes.

Each phase requires regulatory approvals, and progression of a project to each new phase is subject to “go/no-go” decision criteria, which depends on efficacy and safety endpoints, regulatory and ethical approvals, as well as community acceptance of the technology (WHO, 2021b). Testing would be suspended or placed on hold if the responsible regulatory authority of the develop makes a “no-go” decision, whilst community acceptance would be a crucial deciding factor for future testing in a given location (WHO, 2021b).

## 4 Conclusion

Following recent stalled progress towards reducing malaria transmission, there have been several developments in malaria control tools. The progress and potential application of gene drive technology bring much-needed fresh hope in the fight against malaria alongside other existing and novel tools such as bed nets and the RTS,S vaccine. For gene drive technology, important lessons have been learned in the development of CRISPR-based homing drives. Some biological considerations for the design of such drives include (but are not limited to) the:

• conservation of the target sequence,
• density dependence effects from targeting a particular developmental stage for population suppression strategies,
• expression profile of the Cas9 protein to minimise parental deposition to the embryo and/or leakiness into somatic tissues, and
• multiplexing gRNAs to reduce the possibility for the selection of gene drive resistant alleles.

We aimed to highlight critical improvements to the overall design of CRISPR/Cas9-based homing drives and recommend crucial considerations for enhanced bioinformatic selection of target sequences that reduce the likelihood of the selection of mutations that could lead to the loss of the gene drive element, and the significance of a close match to improve the likelihood of mating between released gene drive mosquitoes and the target population in the wild.

We also discussed the need for the further development, rigorous testing, risk assessments and regulatory approval of the various anti-drive systems and approaches for the control of gene drive elements prior to their release. These strategies have the potential to help mitigate any potential predicted or unforeseen risks, however, research is still at an early stage. Much remains to be done to ensure gene drives products are ready for implementation, such as enhancing gene drive efficiency in the lab to maximise the possibility for success in the field and minimise the risk of developing gene drive resistance to acceptable levels. In addition, as for all new vector management technologies, the need remains for continuous knowledge-exchange in relation to all aspects and stages of gene drive development, testing and implementation.
