# Supplementary material for: Driving down malaria transmission with engineered gene drives
Source: Front Genet. 2022 Oct 19;13:891218. doi: 10.3389/fgene.2022.891218 (PMC9627344; doi:10.3389/fgene.2022.891218)
Supplement: Supplementary file 1 [file Table1.DOCX]

| Term | Definition | Definition quoted from | Term reference |
| --- | --- | --- | --- |
| CRISPR (clustered regularly interspaced short palindromic repeats)/Cas9 (CRISPR-associated protein 9) | CRISPR plays a major role in the immunity of bacteria and archaea against viruses. The system uses CRISPR-associated (Cas) nucleases and short RNA sequences, 17 to 20 bp long, to recognise and digest DNA that enters the microbial cell that are complementary to the CRISPR sequence. Together, CRISPR and Cas9 enzyme sequences (CRISPR/Cas9) can be used to edit genes within organisms. |  | (Cong *et al.*, 2013) |
| CRISPR-based Homing drives | Homing drives that encode an endonuclease (such as CRISPR/Cas9) that recognises and cuts a target DNA sequence of ~12-40bp in the host genome, which when repaired by homology-directed repair (HDR) using the gene drive allele as a template, will convert a hemizygous cell into one that is homozygous for the gene drive. |  | (DiCarlo *et al.*, 2015; Gantz and Bier, 2015; Hammond *et al.*, 2016) |
| Daisy chain drives | A multi-component form of homing drive in which multiple split drives are linked into a chain such that each can exhibit homing and hence biased inheritance (drive) only in the presence of the previous element in the chain. The first element in the chain does not drive; this limits the geographic spread and temporal persistence of the drive components, while allowing more rapid spread ("stronger drive") of the later elements of the chain than would a similar two-component split drive system. Several variants of this system have been described and modelled, including 'daisyfield', with multiple parallel components replacing elements of the linear 'chain' and combinations of daisy drive with underdominance-based systems ('daisy quorum'). | (Alphey *et al.*, 2020) | (Noble *et al.*, 2019) |
| Gene drive | ﻿“Gene drive” is used both to describe a process or phenomenon (the biological activity of gene drive) and to describe an object (“a gene drive”). The term sometimes is also used to describe a management tool or intent for product development or regulatory purposes.  I. Process or Phenomenon: A gene drive is a phenomenon of biased inheritance in which the prevalence of a genetic element (natural or synthetic) or specific alternate form of a gene (allele) is increased, even in the presence of some fitness cost. This leads to the preferential increase of a specific genotype that may determine a specific phenotype from one generation to the next and potentially spread throughout a population. In other words, a gene drive is a process that pro- motes or favours the biased inheritance of certain genes from generation to generation.  II. Material Object: A gene drive is composed of one or more genetic elements that can cause the process of biased inheritance in its favour. The set of necessary elements may be referred to as a gene drive system or simply a “gene drive.” Note that the presence of gene drive elements will not necessarily cause gene drive — many gene drive systems will cause the gene drive phenomenon only under specific circumstances (*e.g*., if they are present in the population above a certain threshold frequency, or if fitness costs are below a certain threshold, or if all drive components are present in the organism). Note also that gene drive, when defined as an object, need not always confer preferential transmission. Gene drives must ensure biased inheritance under at least some circumstances but not necessarily all circumstances. For example, some gene drive systems confer preferential inheritance only when present in the population above a threshold frequency. That is, a gene drive is any genetic element able to bias its inheritance within a population.  III. Intention: A gene drive may be intended as a management tool to achieve a particular goal. A gene drive may include additional “cargo” elements, in addition to the drive components, that are intended to introduce new trait(s) into an interbreeding population so as to effect a change in the characteristics of the population. A gene drive also may cause effects directly, for example by inserting into and disrupting a target gene. Thus, a gene drive is a tool to effect certain changes in a population.  See (Alphey *et al.*, 2020) for in depth discussion on important caveats. | (Alphey *et al.*, 2020) | (Sandler and Novitski, 1957) first coined the term “meiotic drive” as a phenomenon of changing allele frequencies in natural populations. |
| High threshold drives | Mathematical modelling shows that many gene drive designs have a 'threshold' level, a ratio of gene-drive-bearing organisms to wild-type organisms in a population, that must be exceeded for the gene drive to be likely to invade that population. This ratio is often modelled in the context of a single release, but the concept applies similarly to continuous releases. For high-threshold drives [such as underdominance-based designs], this ratio is relatively high. High-threshold drives are relatively unlikely to efficiently invade a population from a small accidental release, or rare migration from an adjacent gene-drive-containing population, for example see also spatially restricted drives. | (Alphey *et al.*, 2020) | (Akbari *et al.*, 2013) |
| Homing | The process by which an endonuclease cleaves a specific DNA target sequence and copies itself, or ‘homes’, into this target sequence. Homing utilizes the cell’s homology-directed repair (HDR) machinery, which relies on sequences that flank the endonuclease and that are homologous to either side of the target sequence. The ultimate result of ‘homing’ is to generate an exact copy of the endonuclease in the target sequence. | (Champer *et al.*, 2016) | (Dujon *et al.*, 1989) |
| Integral gene drives (IGDs) | In contrast to the design of conventional population replacement constructs consisting of transgenic promoter- and terminator-driven gene cassettes, IGDs integrate the endonuclease coding sequence (*e.g.*, Cas9) directly within an endogenous gene, the function and expression of which is confined to the male and/or female germline where homing occurs. | Definition modified from (Nash *et al.*, 2018) | (Nash *et al.*, 2018) |
| Integrated Vector Management | A rational decision-making process that encourages optimal use of resources for efficient, cost-effective and sustainable vector control. | (WHO, 2022) | (WHO, 2012) |
| Introgression | The transfer of genetic information from one population to another as a result of hybridization between them and repeated backcrossing. | Definition modified from (Alphey *et al.*, 2020) | Original definition: (Anderson and Hubricht, 1938) |
| Killer-rescue | A temporally self-limiting toxin-antidote-based gene drive system in which both toxin and antidote act zygotically and are encoded at different genetic loci. The toxin gene (Killer, K) imposes a relative fitness disadvantage on non-carriers of the antidote gene (Rescue, R) and can thereby lead to the antidote gene increasing in frequency in the target population. The magnitude of the fitness cost to R- individuals, and hence the relative fitness advantage of Rescue carriers (R+) depends on the penetrance and allele frequency of K. Since K does not drive and has a severe fitness cost in the absence of R, K will tend to decrease in frequency quite rapidly, so the drive property of R is transient. | (Alphey *et al.*, 2020) | (Gould *et al.*, 2008) |
| Population replacement (or population modification/conversion/alteration) | Strategies that target vector competence with the intent to reduce the inherent ability of individual vectors to transmit a given pathogen. Sometimes referred to as "population replacement" or "population conversion" or 'alteration' | (Alphey *et al.*, 2020) | (WHO, 1964) |
| Population suppression | Strategies that target “demography” with the intent to reduce (suppress) the size of a natural population. For vector populations, the intent may be to reduce (suppress) the size of the target vector population to the extent that it would not be able to sustain pathogen transmission (R0<1) | (Alphey *et al.*, 2020) | (WHO, 1964) |
| Private alleles | Alleles that are found only in a single population among a broader collection of populations. | (Szpiech and Rosenberg, 2011) | (Szpiech and Rosenberg, 2011) |
| RIDL (Release of insects carrying a dominant lethal) | A form of SIT (sterile insect technique) that relies on the release of insects that carry a dominant lethal gene. The lethal gene likely needs to be conditional (repressible) in order to 'turn off' the lethality in order to allow the strain to be reared (at all, but especially in the large numbers typical of SIT programmes). | (Alphey *et al.*, 2020) | (Thomas *et al.*, 2000) |
| Self-sustaining gene drive | Self-sustaining gene drives are designed to cause specific sequences to increase in frequency in a target population; in some cases, the sequences may become fixed in the population. The specific sequences will normally include part or all of the gene drive and may include additional sequences (*e.g.,* cargo genes). In the absence of mutation or heritable resistance, these drives can potentially sustain the high frequency of these sequences indefinitely in the target population | Definition modified from (Alphey *et al.*, 2020) | (WHO, 2014) |
| Self-limiting gene drive | Approaches where the genetic modification will not pass on indefinitely through subsequent generations, even in the absence of mutation or heritable resistance. For gene drives, this will typically mean that the allele frequency of the gene drive will initially increase (the gene drive phenomenon) but later decrease, eventually disappearing. Also known as self-exhausting drives or approaches. Examples of self-limiting drives include Split drives, daisy drives and Killer-Rescue | (Alphey *et al.*, 2020) | (WHO, 2014) |
| Spatially restricted (or localised) drives | Also known as local drives or partially restricted gene drives. These are gene drives that are geographically confined. Potential means to restrict spread of drives include use of high-threshold drives and/or self-limiting drives | Definition modified from (Alphey *et al.*, 2020) | (Friedman *et al.*, 2020) |
| Split drives | In a split gene drive, the necessary components for gene drive are split between two (or more) genetic loci. If one (or more) of these components is incapable of gene drive even in the presence of the others, this is a non-autonomous gene drive. If, in the presence of the other elements, each element can exhibit gene drive ("trans-complementing split gene drive") the components in suitable combination may comprise an autonomous gene drive. | (Alphey *et al.*, 2020) | (Dhole *et al.*, 2019; Edgington *et al.*, 2020) |
| Sterile Insect Technique (SIT) | The SIT is a genetic control method that involves the overflooding of a target population with sterile insects, resulting in the reduction of the proportion of productive matings and the eventual reduction in the size of the receiving population. Sterile males are generally thought to be the key control element; some SIT programmes aim to release only males. Males may be sterilised by any of several methods, including radiation, chemo-sterilisation, or genetic modification. | Definition modified from (Alphey *et al.*, 2020) | (Knipling, 1955, 1979) |
| Temporally self-limiting | Drive systems in which the system is predicted to persist for a finite time in the target population or species. This is taken to mean (i) after releases cease, since periodic releases could sustain presence indefinitely and (ii) even in the absence of mutation or heritable resistance, since genetic change of this type may lead to any genetic system being unstable over time. It is also assumed that the drive system has not become fixed in the population. | (Alphey *et al.*, 2020) | (Dhole *et al.*, 2018) |
| Tethered drive | Also known as tethered homing gene drives. These drives include a homing component that does not drive on its own, but is “tethered” by engineering it to be reliant on a spatially restricted gene drive. For example, a tethered homing drive could use a two-locus engineered underdominance component to tether a CRISPR/Cas-based homing component (underdominance tethered homing, UTH). Conceptually, the homing component can instead be tethered to a different localized gene drive, such as one-locus engineered underdominance, chromosomal translocations, or one of the toxin–antidote systems. | Definition modified from (Dhole *et al.*, 2019) | (Dhole *et al.*, 2019) |
| Toxin-antidote-based drive system | A diverse set of systems, including a range of naturally occurring gene drive systems, in which a cell or organism deposits a toxin into most or all of its offspring such that those that do not inherit an antidote gene have a significant fitness cost. This can lead to preferential inheritance of the antidote gene. | (Alphey *et al.*, 2020) | (Marshall, 2011; Marshall *et al.*, 2011; Marshall and Hay, 2011) |
| Underdominance | The unusual situation where a heterozygote shows an attribute, such as viability or fertility, that is lower than in either homozygote. For example, the New Zealand Black (NZB) strain of mouse spontaneously develops a disease that resembles lupus erythematosis in humans. New Zealand White (NZW) mice are normal in this regard. The hybrid offspring from crossing these inbred strains of mice (NZB x NZW) develop a more severe disease than that of the NZB strain. Underdominance in classical genetics refers to a situation at a single locus where a heterozygote for two different alleles has lower fitness than either of the two homozygotes. In gene drive parlance the concept is expanded to consider two true-breeding strains and their F1 hybrid: if the hybrid is of lower fitness than either of the true-breeding parental types this is underdominance. This is the converse of the better-known phenomenon of hybrid vigour, wherein the hybrid has higher fitness than either of the parental types. | (Alphey *et al.*, 2020) | (Davis *et al.*, 2001) |

**References**

Akbari, O.S., Matzen, K.D., Marshall, J.M., Huang, H., Ward, C.M. and Hay, B.A. (2013), “A synthetic gene drive system for local, reversible modification and suppression of insect populations”, *Current Biology*, Vol. 23 No. 8, pp. 671–677.

Alphey, L.S., Crisanti, A., Randazzo, F. and Akbari, O.S. (2020), “Standardizing the definition of gene drive”, *PNAS*, Vol. 117 No. 49, pp. 30864–30867.

Anderson, E. and Hubricht, L. (1938), “Hybridization in Tradescantia. III. The Evidence for Introgressive Hybridization”, *American Journal of Botany*, Vol. 25 No. 6, p. 396.

Champer, J., Buchman, A. and Akbari, O.S. (2016), “Cheating evolution: engineering gene drives to manipulate the fate of wild populations”, available at:https://doi.org/10.1038/nrg.2015.34.

Cong, L., Ran, F.A., Cox, D., Lin, S., Barretto, R., Habib, N., Hsu, P.D., *et al.* (2013), “Multiplex genome engineering using CRISPR/Cas systems.”, *Science (New York, N.Y.)*, Science, Vol. 339 No. 6121, pp. 819–23.

Davis, S., Bax, N. and Grewe, P. (2001), “Engineered underdominance allows efficient and economical introgression of traits into pest populations”, *Journal of Theoretical Biology*, Vol. 212 No. 1, pp. 83–98.

Dhole, S., Lloyd, A.L. and Gould, F. (2019), “Tethered homing gene drives: A new design for spatially restricted population replacement and suppression”, *Evolutionary Applications*, John Wiley & Sons, Ltd, Vol. 12 No. 8, pp. 1688–1702.

Dhole, S., Vella, M.R., Lloyd, A.L. and Gould, F. (2018), “Invasion and migration of spatially self-limiting gene drives: A comparative analysis.”, *Evolutionary Applications*, Evol Appl, Vol. 11 No. 5, pp. 794–808.

DiCarlo, J.E., Chavez, A., Dietz, S.L., Esvelt, K.M. and Church, G.M. (2015), “Safeguarding CRISPR-Cas9 gene drives in yeast.”, *Nature Biotechnology*, NIH Public Access, Vol. 33 No. 12, pp. 1250–1255.

Dujon, B., Beifort, M., Butow, R.A., Jacq, C., Lemieux, C., Perlman, P.S. and Vogt, V.M. (1989), “Mobile introns: definition of terms and recommended nomenclature”, *Gene*, Elsevier, Vol. 82 No. 1, pp. 115–118.

Edgington, M.P., Harvey-Samuel, T. and Alphey, L. (2020), “Split drive killer-rescue provides a novel threshold-dependent gene drive”, *Scientific Reports*, Nature Publishing Group, Vol. 10 No. 1, p. 20520.

Friedman, R.M., Marshall, J.M. and Akbari, O.S. (2020), “Gene Drives New and Improved”, *Issues in Science & Technology*, Vol. Winter, pp. 72–78.

Gantz, V.M. and Bier, E. (2015), “The mutagenic chain reaction: A method for converting heterozygous to homozygous mutations”, *Science*, Vol. 348 No. 6233, pp. 442–444.

Gould, F., Huang, Y., Legros, M. and Lloyd, A.L. (2008), “A Killer-Rescue system for self-limiting gene drive of anti-pathogen constructs”, *Proceedings of the Royal Society Biological Sciences*, Vol. 275 No. 1653, pp. 2823–2829.

Hammond, A., Galizi, R., Kyrou, K., Simoni, A., Siniscalchi, C., Katsanos, D., Gribble, M., *et al.* (2016), “A CRISPR-Cas9 gene drive system targeting female reproduction in the malaria mosquito vector *Anopheles gambiae*”, *Nature Biotechnology*, Vol. 34 No. 1, available at:https://doi.org/10.1038/nbt.3439.

Knipling, E.F. (1955), “Possibilities of insect control or eradication through the use of sexually sterile males”, *Journal of Economic Entomology*, Vol. 48 No. 4, pp. 459–462.

Knipling, E.F. (1979), *The Basic Principles of Insect Population and Suppression and Management*, United States Department of Agriculture, Washington, USA.

Marshall, J.M. (2011), “The toxin and antidote puzzle: new ways to control insect pest populations through manipulating inheritance.”, *Bioengineered Bugs*, Bioeng Bugs, Vol. 2 No. 5, pp. 235–40.

Marshall, J.M. and Hay, B.A. (2011), “Inverse Medea as a novel gene drive system for local population replacement: a theoretical analysis.”, *The Journal of Heredity*, J Hered, Vol. 102 No. 3, pp. 336–41.

Marshall, J.M., Pittman, G.W., Buchman, A.B. and Hay, B.A. (2011), “Semele: a killer-male, rescue-female system for suppression and replacement of insect disease vector populations.”, *Genetics*, Genetics, Vol. 187 No. 2, pp. 535–51.

Nash, A., Urdaneta, G.M., Beaghton, A.K., Hoermann, A., Papathanos, P.A., Christophides, G.K. and Windbichler, N. (2018), “Integral gene drives for population replacement”, *Biology Open*, The Company of Biologists, Vol. 8 No. 1, available at:https://doi.org/10.1242/bio.037762.

Noble, C., Min, J., Olejarz, J., Buchthal, J., Chavez, A., Smidler, A.L., DeBenedictis, E.A., *et al.* (2019), “Daisy-chain gene drives for the alteration of local populations.”, *Proceedings of the National Academy of Sciences of the United States of America*, National Academy of Sciences, Vol. 116 No. 17, pp. 8275–8282.

Sandler, L. and Novitski, E. (1957), “Meiotic Drive as an Evolutionary Force”, *The American Naturalist*, Science Press , Vol. 91 No. 857, pp. 105–110.

Szpiech, Z.A. and Rosenberg, N.A. (2011), “On the size distribution of private microsatellite alleles.”, *Theoretical Population Biology*, NIH Public Access, Vol. 80 No. 2, pp. 100–13.

Thomas, D., Donnelly, C.A., Wood, R.J. and Alphey, L. (2000), “Insect Population Control Using a Dominant, Repressible, Lethal Genetic System”, *Science*, Vol. 287 No. 5462, pp. 1476–2474.

WHO. (1964), *Genetics of Vectors and Insecticide Resistance*, available at: https://apps.who.int/iris/bitstream/handle/10665/40573/WHO_TRS_268.pdf?sequence=1 (accessed 7 September 2022).

WHO. (2012), *HANDBOOK for Integrated Vector Management Integrated Vector Management (IVM) Vector Ecology and Management (VEM) Department of Control of Neglected Tropical Diseases (NTD) World Health Organization*, available at: https://apps.who.int/iris/bitstream/handle/10665/44768/9789241502801_eng.pdf;jsessionid=56DEE391B6CCFCD9978A09CC348E7DB5?sequence=1 (accessed 7 September 2022).

WHO. (2014), *Guidance Framework for Testing of Genetically Modified Mosquitoes*, Geneva, Switzerland.

WHO. (2022), “Integrating vector management”, available at: https://www.who.int/westernpacific/activities/integrating-vector-management (accessed 21 July 2022).
